# Late testicular relapse two decades after primary extragonadal germ cell tumor with uncommon metastases: a case report

**DOI:** 10.1186/s13256-021-02667-y

**Published:** 2021-02-05

**Authors:** Motoi Tobiume, Shigeyuki Aoki, Genya Nishikawa, Hiroyuki Muramatsu, Kenzo Ono, Shingo Morinaga, Koji Hara, Natsuko Ando, Keiko Ono, Chie Nishibata, Ayumi Hidano, Makiko Nakagawa, Ikumi Takahashi, Kanae Matsubara, Yoshiaki Yamada

**Affiliations:** 1 The Department of Urology, Japan Organization of Occupational Health and Safety Asahi Rosai Hospital, 61 Hirakocho, Owariasahi-shi, Aichi 488-8585 Japan; 2 The Department of Urology, Japan Community Health care Organization Kani Tono Hospital, 1221-5 Dota, Kani-shi, Gifu 509-0206 Japan; 3 The Department of Pathology, Japan Organization of Occupational Health and Safety Asahi Rosai Hospital, 61 Hirakocho, Owariasahi-shi, Aichi 488-8585 Japan; 4 The Division of Nursing, Japan Organization of Occupational Health and Safety Asahi Rosai Hospital, 61 Hirakocho, Owariasahi-shi, Aichi 488-8585 Japan; 5 The Division of Nursing, Japan Community Health care Organization Kani Tono Hospital, 1221-5 Dota, Kani-shi, Gifu 509-0206 Japan

**Keywords:** Extragonadal germ cell tumor, Metachronous testicular tumor, Seminoma

## Abstract

**Background:**

Extragonadal germ cell tumor (EGCT) is a relatively rare condition, reportedly representing 3–7% of all germ cell tumors. We report a patient who had metachronous testicular tumor with uncommon metastases 20 years after primary retroperitoneal EGCT treatment, along with a corresponding literature review.

**Case presentation:**

A 49-year-old Japanese man visited our department in November 2017 with chief complaints of indolent right scrotum enlargement and a right inguinal mass. History showed that the patient visited our department of gastroenterology with chief complaints of blackish feces and ill complexion in February 1997. Computed tomography (CT) showed a right retroperitoneal tumor, which was removed in the same month. Histopathological examination showed a teratoma and yolk sac tumor. He was diagnosed with primary retroperitoneal EGCT and received three courses of chemotherapy (bleomycin/etoposide/cisplatin; BEP). Periodic imaging and the determination of tumor markers (alpha-fetoprotein [AFP], human chorionic gonadotropin [HCG], and lactate dehydrogenase [LDH]) showed no recurrence or metastasis during the 5 years postoperatively. Subsequently, he did not visit the outpatient ward. In August 1999, he underwent surgery of right hydrocele. Contrast-enhanced CT showed a 35-mm contrast effect with uneven content in the right testicle and enlarged nodes that raised suspicion for metastases in the right inguinal and right external iliac lymph nodes. All tumor markers were within normal ranges. He underwent right high orchiectomy and resection of the right inguinal lymph nodes in the same month. Histopathological findings revealed seminoma (pT1, pN2, M0, S0, and TNM stage IIB). He received postoperative chemotherapy, one course of BEP therapy, and three courses of etoposide and cisplatin therapy. Post-chemotherapy CT confirmed a complete clinical response at the right external iliac lymph nodes, and this response continued 12 months later. No recurrence or metastasis has been found so far.

**Conclusions:**

We report a patient in whom a testicular tumor with uncommon metastases occurred 20 years after primary retroperitoneal EGCT treatment. After EGCT treatment, testicular relapses tend to occur after relatively long-term follow-up. After EGCT treatment, such patients must be closely monitored for testicular recurrences and onset of testicular tumor.

## Background

The primary site of male germ cell tumors is most commonly the testicle. Extratesticular (extragonadal) onset of these tumors is reported in 3–7% of cases. It has been reported that metachronous testicular tumors may occur after the treatment of extragonadal germ cell tumor (EGCT) in 5% of EGCT patients [1]. This case report describes a patient who experienced a testicular tumor with uncommon metastases 20 years after primary retroperitoneal EGCT treatment, along with a corresponding literature review.

## Case presentation

A 49-year-old Japanese man visited our department in November 2017 with chief complaints of indolent right scrotum enlargement and a right inguinal mass. History showed that the patient visited our department of gastroenterology with chief complaints of blackish feces and ill complexion in February 1997. Gastrointestinal fiberscopy was used to treat duodenal ulcer bleeding. Computed tomography (CT) showed a right retroperitoneal tumor, which was removed in the same month.

**Fig. 1:**
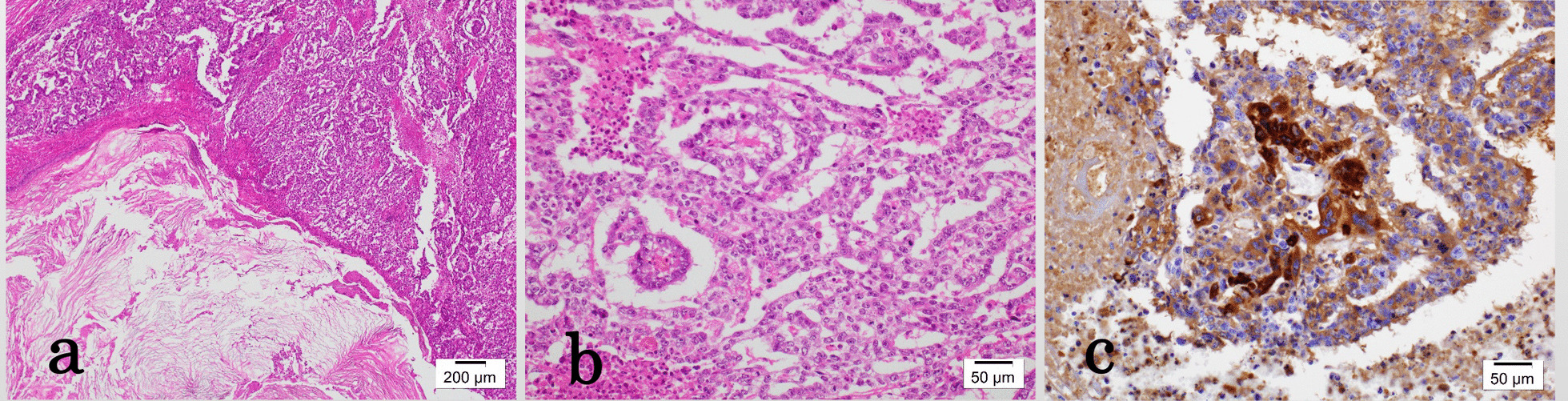
Histopathological findings of the primary retroperitoneal extragonadal germ cell tumor show the characteristics of teratoma for hematoxylin and eosin (HE) staining (**a** ×100) and yolk sac tumor for HE staining (**b** ×200); the site of the yolk sac tumor was positive for alpha-fetoprotein (AFP) immunostaining (**c** ×200).

Histopathological examination showed a teratoma and yolk sac tumor (Fig. 1). He was diagnosed with primary retroperitoneal EGCT and underwent three courses of chemotherapy (bleomycin/etoposide/cisplatin; BEP) starting in May 1997. He was closely followed using procedures including periodic imaging, tumor markers determination, and self-palpation.

He was followed up on an outpatient basis 5 years after treatment and was subsequently lost to follow-up. In August 1999, he underwent surgery of the right hydrocele. At that time, perioperative findings were unremarkable, indicating no clear tumor in either testicle.

At the first visit, his height was found to be 163 cm, and weight was 58.5 kg.

Blood pressure was 128/78 mmHg, heart rate was regular at 62 beats per minute, and body temperature was 36.4 °C.

He had no history of smoking or drinking, and there was nothing remarkable in the family history.

On physical examination, the right testicle was found to be elastic, hard, and enlarged to the size of 40 × 40 mm. A 45 × 40 mm sized induration was palpable in the right inguinal area and was adequately mobile. Neurological examination showed no abnormal findings. Blood biochemistry examination, urinalysis, and tumor markers (alpha-fetoprotein [AFP], human chorionic gonadotropin [HCG], and lactate dehydrogenase [LDH]) showed no abnormal findings.

Ultrasound findings showed a mosaic shadow inside the right testicle, with no abnormal findings in the left testicle.

**Fig. 2:**
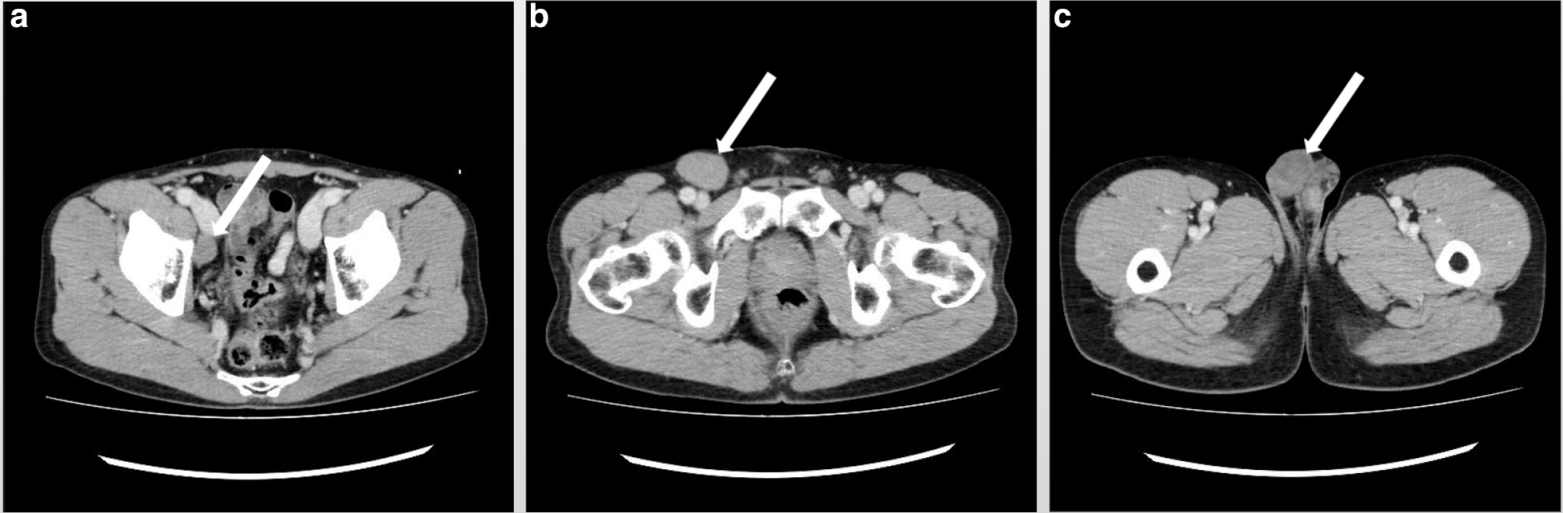
Contrast-enhanced pelvic CT. **a** Enlarged right external iliac lymph node (white arrow). **b** Enlarged right inguinal lymph node (white arrow). **c** Enhanced right testicular tumor (white arrow). The diagnosis was testicular tumor (cT1, N2, M0, S0, and TNM stage IIB)

During other diagnostic imaging procedures, enlargements of the right external iliac lymph node (24 × 14 mm) and right inguinal lymph node (43 × 29 mm) and a 31 × 22 mm mass with uneven contents were found in the right testicle (Fig. 2). There was no evidence of any distant metastasis.

**Fig. 3:**
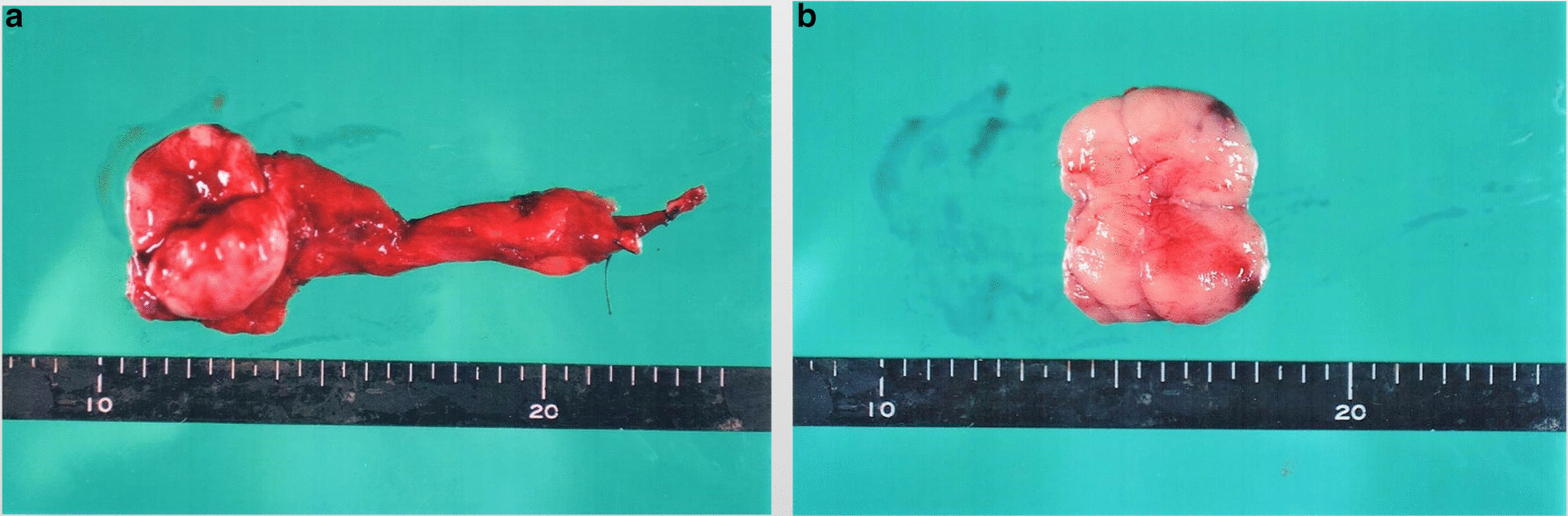
**a** Resected testicular tumor was 40 × 40 × 30 mm in size and weighed 34 g. **b** Resected inguinal lymph node was 40 × 40 × 30 mm in size and weighed 21 g. The cut surfaces of both specimens were yellowish and solid, and the testicular tumor was localized in the testicle.

For the right testicular tumor, the patient underwent high orchiectomy and resection of the right inguinal lymph nodes in November 2017. The testicular tumor was 40 × 40 × 30 mm in size and weighed 34 g. The lymph node was 40 × 40 × 30 mm in size and weighed 21 g. The cut surfaces of both specimens were yellowish and solid, and the testicular tumor was localized in the testicle (Fig. 3).

**Fig. 4:**
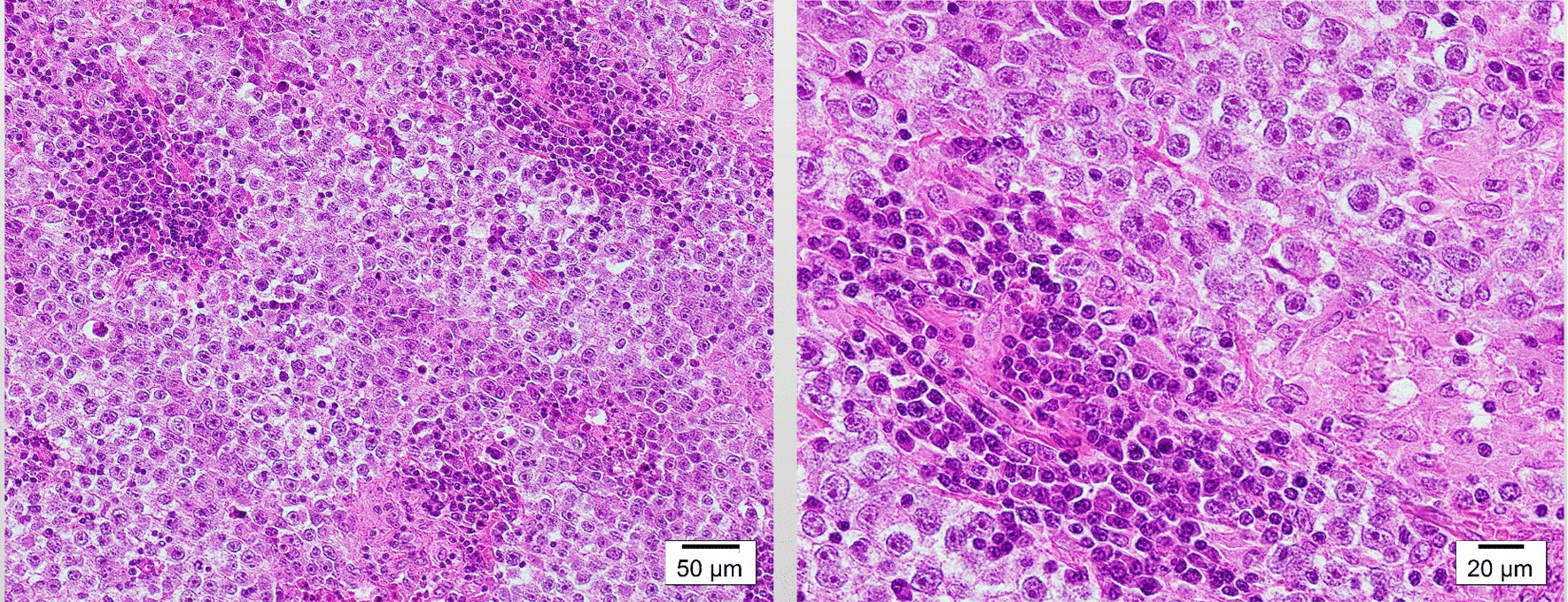
Both the testicular tumor and inguinal lymph node presented with irregular cobblestone proliferations of germ cell-like atypical cells with clear nucleoli and no vascular proliferation. Spermatic cord stump was negative, showing that this tumor was a classical seminoma for hematoxylin and eosin staining (left ×100, right ×200).

Histopathological findings showed irregular cobblestone proliferations of germ cell-like atypical cells with clear nucleoli and massive necrotic changes that partially resulted from self-destruction. The tumor did not extend into the tunica albuginea, and no vascular infiltration was found. Spermatic cord stumps were negative. Tumor diagnosis was a classical seminoma with no other elements. Lymph node metastases were also diagnosed as a result of the seminoma (Fig. 4).

Based on the above findings, this seminoma was diagnosed as pT1, pN2, M0, S0, and TNM stage IIB [2].

The patient received postoperative chemotherapy with one course of BEP therapy and three courses of etoposide and cisplatin (EP) therapy at the Japan Community Health care Organization Kani Tono Hospital starting in January 2018.

**Fig. 5:**
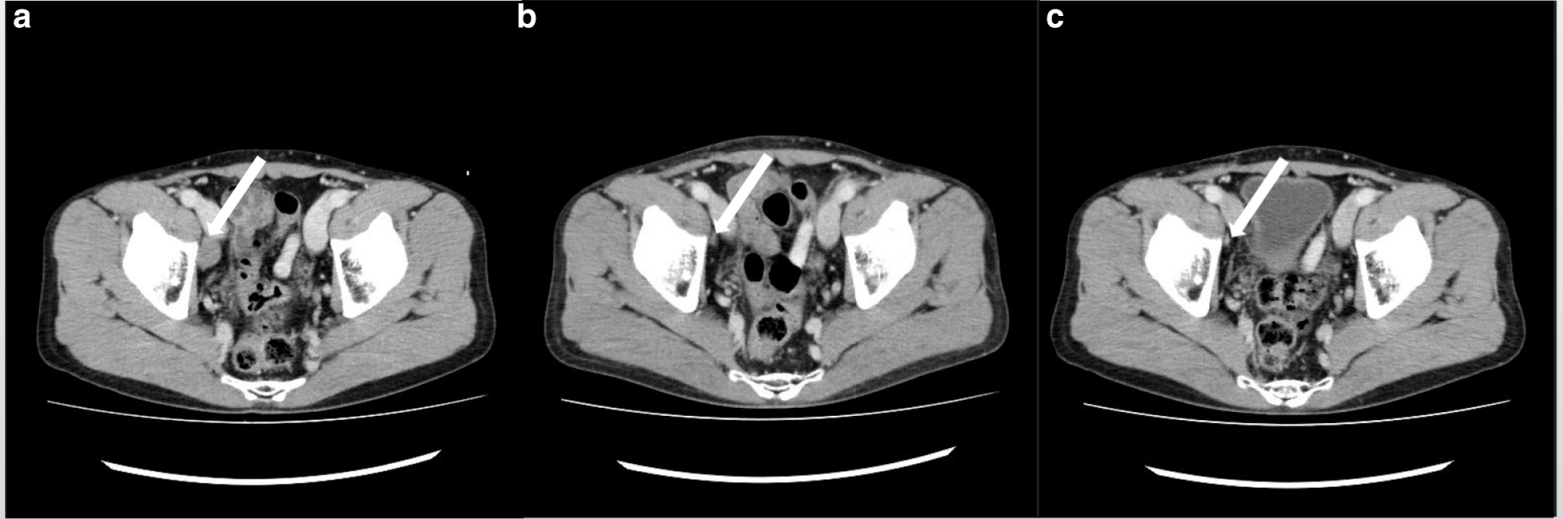
Contrast enhanced pelvic CT images before chemotherapy. **a** Enlarged right external iliac lymph node (white arrow). **b** Clinical complete response was confirmed after chemotherapy (white arrow). **c** Clinical complete response continued 12 months after chemotherapy (white arrow)

Post-chemotherapy CT confirmed a complete clinical response (cCR) at the right external iliac lymph node, and this response continued 12 months later (Fig. 5).

## Discussion

Metastases of the testicular tumor to the retroperitoneal lymph node are the most common. We reported a patient who had metachronous testicular tumor with metastases to the groin and external iliac lymph nodes 20 years after primary retroperitoneal EGCT treatment.

EGCT accounts for 3–7% of all germ cell tumors [1]. Several onset mechanisms have been reported; primordial germ cells remain at extragonadal sites, including the mediastinum and retroperitoneum, due to the abnormal migration of such cells during mobilization to the urogenital swelling at embryogenesis, and EGCT originates from germ cells that are physiologically distributed in the liver, bone marrow, and brain for the transport of hematological and immunological information [3, 4]. Among patients who experienced cCR, metachronous testicular tumor (MTT) was reported in approximately 5% of patients [1]. Among patients who experienced cCR after EGCT treatment, MTT onset was reported in 16 of 635 patients as reported by Bokemeyer *et al*. [5], and 5 of 51 patients as reported by Hashimoto *et al*. [6].

The mechanism of MTT after EGCT is unknown. Daugaard *et al*. performed bilateral testicular biopsy in 46 patients who were diagnosed with EGCT after excluding testicular tumors by palpation and ultrasound examinations [7]. Germ cell neoplasia *in situ* (GCNIS) was confirmed in 42% of the patients, and the origins of EGCT and GCNIS were reported to be the same. The patient in this report, however, received three courses of BEP therapy after EGCT diagnosis. It was impossible to establish why chemotherapy was administered. Our patient experienced cCR after BEP and EP therapies, similar to his treatment 20 years previously. If the origin was the same between EGCT and GCNIS, the latter tumor must have disappeared after the previous chemotherapy. We therefore did not consider that the origins of EGCT and GCNIS were the same.

Tanio *et al*. summarized 37 cases of MTT after EGCT treatment in Japan and overseas countries [8]. After exclusion of four patients whose primary sites were unknown and the mediastinum, the most frequent site was the retroperitoneum, as reported in 29 patients. According to our search of patients with primary retroperitoneal EGCT, including the patient in this article, MTT was found in 31 patients. Table 1 summarizes these patients [1, 6, 9–18]. The age of the patients ranged from 18 to 49 years, with a median age of 30 years. In most patients, the histological type was non-seminoma for EGCT and seminoma for MTT. The histological type in our patient also differed from previous reports. The time to MTT onset was relatively long, ranging from 14 to 248 months, with a median of 64 months, and the time to the second onset 20 years after treatment in our patient was the longest among the 31 patients. At the time of MTT diagnosis, 18 patients had no metastasis, and seven patients, including our patient, experienced metastases. All patients underwent a high orchiectomy. Patients with metastases received postoperative chemotherapy. Prognosis was satisfactory; among patients whose postoperative courses were available, 28 patients were alive, with no recurrence in all but one patient.

**Table 1:** Summary of previously reported cases of patients with retroperitoneal extragonadal germ cell tumor who later developed metachronous testicular tumor

Case	Author	Age	EGCT histological type	MTT developmental period (month)	MTT histological type	MTT metastasis at diagnosis	Additional treatment	Recurrence
1	Quintela [9]	44	sem	84	non-sem	–	–	–
2	Lokich [10]	22	non-sem	168	sem	–	–	Unknown
3	Hayashi [11]	18	non-sem	84	sem	–	–	–
4	Gerl [12]	Unknown	non-sem	35	sem	–	–	–
5	Gerl [12]	Unknown	non-sem	42	sem	–	–	–
6	Gerl [12]	Unknown	non-sem	77	sem	+	Chemotherapy	–
7	Allaway [13]	22	non-sem	84	sem	+	Chemotherapy	–
8	Daniel [14]	24	sem	60	non-sem	–	–	After 3 months
9	Daniel [14]	23	non-sem	23	non-sem	Unknown	Radiation therapy	–
10	Hartmann [1]	28	non-sem	74	sem	+	Chemotherapy	–
11	Hartmann [1]	33	non-sem	30	non-sem	+	Chemotherapy	–
12	Hartmann [1]	40	non-sem	100	sem	+	Chemotherapy	–
13	Hartmann [1]	49	non-sem	88	sem	–	–	–
14	Hartmann [1]	29	non-sem	30	non-sem	–	–	–
15	Hartmann [1]	22	non-sem	48	sem	–	–	–
16	Hartmann [1]	23	non-sem	14	sem	–	–	–
17	Hartmann [1]	34	non-sem	35	sem	–	–	–
18	Hartmann [1]	Unknown	non-sem	102	sem	–	–	–
19	Hartmann [1]	30	non-sem	42	sem	+	Chemotherapy	–
20	Hartmann [1]	34	non-sem	78	sem	–	–	–
21	Hartmann [1]	22	non-sem	39	non-sem	–	–	–
22	Mindrup [15]	42	non-sem	50	non-sem	–	–	Unknown
23	kuroda [16]	32	non-sem	48	sem	–	–	–
24	Yamada [17]	30	non-sem	96	sem	–	–	–
25	Kawamura [18]	33	Unknown	91	non-sem	–	–	–
26	Hashimoto [6]	27	non-sem	120	sem	Unknown	Unknown	–
27	Hashimoto [6]	30	sem	96	sem	Unknown	Unknown	–
28	Hashimoto [6]	38	non-sem	64	non-sem	Unknown	Unknown	–
29	Hashimoto [6]	32	non-sem	15	sem	Unknown	Unknown	–
30	Hashimoto [6]	47	non-sem	21	non-sem	Unknown	Unknown	–
31	Present case	49	non-sem	248	sem	+	Chemotherapy	–

*EGCT* extragonadal germ cell tumor, *MTT* metachronous testicular tumor, *sem* seminoma

Conventionally, lymph ducts from the testicles flow into the para-aortic and para-vena cava lymph nodes via the spermatic cord. Metastases to the retroperitoneal lymph node were the most frequent, as reported in 88% of EGCT patients [19]. Our patient, however, experienced metastases to the groin and external iliac lymph nodes. Jerome *et al*. reported the following possible reasons for the occurrence of testicular tumor non-regional metastases to groin lymph nodes: (1) tumor infiltration to the epididymis, spermatic cord, and scrotal skin; (2) history of surgical procedures in the groin or scrotum; (3) retrograde metastases from metastases to the retroperitoneal lymph nodes; and (4) direct infiltration of residual lesions in the spermatic cord [20]. In the Japanese General Rules for the Surgical and Pathological Studies on Testicular Tumors, it is noted that in patients undergoing scrotal and groin surgical procedures, lymph nodes in the pelvis and groin are also regarded as regional lymph nodes [21]. Our patient also had a history of radical right hydrocele treatment 18 years previously. This treatment changed his lymph flow, probably causing metastases to the groin and external iliac lymph nodes. In the treatment of patients with testicular tumors who have undergone surgical treatment in the groin or scrotum, it is necessary to consider possible metastases to lymph nodes in the groin and pelvis.

As described previously, MTT prognosis tends to be satisfactory in patients with primary retroperitoneal EGCT. This is because seminoma is the most frequent type of MTT. Moreover, MTT tends to occur after relatively long periods of time and may be a biologically less malignant germ cell tumor. For MTT treatment, it is nevertheless important to consider the fact that MTT may occur 20 years after EGCT treatment, and that the tumor has already metastasized at its diagnosis, as in our patient.

## Conclusion

We encountered a patient in whom a testicular tumor with uncommon metastases occurred 20 years after primary retroperitoneal EGCT treatment. After EGCT treatment, testicular relapses tend to occur after relatively long-term follow-up. After EGCT treatment, such patients must be closely monitored for testicular recurrences and onset of testicular tumor.

## Data Availability

Not applicable.

## Abbreviations

EGCT: Extragonadal germ cell tumor
MTT: Metachronous testicular tumor
CT: Computed tomography
AFP: Alpha-fetoprotein
HCG: Human chorionic gonadotropin
LDH: Lactate dehydrogenase
EP: Etoposide and cisplatin
cCR: Complete clinical response
GCNIS: Germ cell neoplasia *in situ*
